# Synergistic Antimicrobial Activity of Vancomycin, Ceftriaxone, and Gentamicin Against *Cutibacterium acnes* Strains: An In Vitro Checkerboard Analysis and In Vivo Interaction with Bioactive Glass Using *Galleria mellonella*

**DOI:** 10.3390/antibiotics14090923

**Published:** 2025-09-12

**Authors:** Mariana Neri Lucas Kurihara, Isabelle Frois Brasil, Mayara Muniz de Andrade Silva, Mauro Jose Salles

**Affiliations:** 1 Special Laboratory of Clinical Microbiology (LEMC), Department of Medicine, Division of Infectious Diseases, Escola Paulista de Medicina (EPM), Universidade Federal de São Paulo (UNIFESP), São Paulo 04024-002, Brazil; mariana.kurihara@unifesp.br (M.N.L.K.); isabelle.brasil@unifesp.br (I.F.B.); mmasilva@unifesp.br (M.M.d.A.S.); 2 Musculoskeletal Infection Group, Department of Orthopedics and Traumatology, Escola Paulista de Medicina (EPM), Universidade Federal de São Paulo (UNIFESP), São Paulo 04024-002, Brazil; 3 Infectious Disease Discipline, Faculdade de Ciências Médicas da Santa Casa de São Paulo, São Paulo 01224-001, Brazil

**Keywords:** *Cutibacterium acnes*, orthopaedic implant-associated infection, antibiotic synergy, bioactive glass S53P4, *Galleria mellonella* model

## Abstract

Background/Objectives: *Cutibacterium acnes* is increasingly recognized as a relevant pathogen in orthopaedic implant-associated infections, yet treatment strategies remain largely empirical. With rising antimicrobial resistance and scarce data on drug interactions, optimizing targeted therapies is essential. This preclinical study investigated the efficacy and synergism of vancomycin (VA), gentamicin (GEN), and ceftriaxone (CTX) against two clinical phylotype IB strains from orthopaedic infections and the reference strain *C. acnes* ATCC 6919, using both in vitro and in vivo models. Methods: Minimum inhibitory concentrations (MICs) were determined using broth microdilution following BrCAST guidelines. Synergistic activity was assessed using the checkerboard assay and interpreted via fractional inhibitory concentration indices (FICIs). The in vivo efficacy of antibiotic combinations with bioactive glass S53P4 (BAG) was evaluated in the *Galleria mellonella* infection model. Results: All *C. acnes* strains exhibited uniformly low MICs. Synergistic activity was observed for CTX combined with GEN in strain 2 (FICI range 0.25–0.37), while partial synergy was detected for CTX with GEN in strain 1 (FICI ≈ 0.56–0.63), and for CTX combined with VA in the ATCC strain (FICI = 0.66). All other combinations demonstrated indifferent interactions. In the *G. mellonella* model, a high bacterial inoculum (OD_600_ of 3.0) was needed to establish an infection. For all strains tested, the use of antibiotics in combination with BAG improved larval survival. For the clinical strains, the combination of CTX + GEN + BAG and BAG alone demonstrated greater efficacy in promoting larval survival. Conclusions: Acombination of a cephalosporin with an aminoglycoside, particularly when incorporated into a biomaterial matrix, enhances antimicrobial activity against both clinical and reference strains of *C. acnes*.

## 1. Introduction

*Cutibacterium acnes* is a dominant component of the human skin microbiota and has long been linked to dermatological conditions, especially acne vulgaris. Current treatments rely heavily on topical and systemic antimicrobials, such as macrolides, tetracyclines, and lincosamides [1]. As a consequence, the emergence of resistant strains has arisen due to the overprescription of these compounds, particularly those carrying the *rpoB* (rifampicin resistance) and *erm(X)* (clindamycin resistance) genes [2,3,4]. While *C. acnes* resistance is not yet classified as a global healthcare threat, rising resistance rates pose challenges for clinical management and highlight the need for alternative or combination antibiotic strategies. Beyond its dermatological relevance, *C. acnes* has also emerged as an opportunistic pathogen in postoperative and orthopedic implant-associated infections.

The pathogenicity of *C. acnes* varies significantly across its phylogenetic groups (IA1, IA2, IB, IC, II, and III), which exhibit differences in gene content, virulence traits, and clinical associations. Notably, phylotypes IB, IC, and III are more frequently implicated in orthopedic device-related infections, underscoring the need for phylotype-specific data to address targeted therapeutic strategies [5,6,7]. In this context, complete eradication of *C. acnes* is neither feasible nor desirable, given its role in maintaining cutaneous microbial homeostasis [8]. Instead, selective modulation is necessary to balance antimicrobial efficacy with microbiota preservation, especially in surgical settings where infection control is critical.

In the orthopedic setting, local antimicrobial delivery systems, including antibiotic-loaded bone cements and bioceramics, are commonly employed. These biomaterials typically incorporate drugs like vancomycin and gentamicin, which are thermally stable and broadly active [9]. Likewise, systemic antibiotics such as ceftriaxone have been the first choice to treat musculoskeletal infections caused by *C. acnes* [10]. Although vancomycin and gentamicin demonstrate some activity against *C. acnes*, they are not considered first-line drugs. In Brazil, for example, susceptibility to ceftriaxone remains high [11].

Despite their clinical relevance, limited studies have investigated potential synergistic interactions between β-lactamsand other drugs, including rifampicin, which seems to have no impact on clinical outcomes [12,13]. Instead, Kusejko et al. (2021) emphasized that successful outcomes in periprosthetic joint infections (PJIs) caused by *C. acnes* were more strongly associated with surgical strategies, such as debridement, antibiotics, and implant retention (DAIR), and the optimization of antibiotic treatment duration, rather than specific antimicrobial combinations [12]. Moreover, modern bioceramics compounds have been shown to inhibit the growth of several species of plankton and biofilm-forming bacteria in combination with antibiotics [13,14]. Bioactive glass S53P4 expresses local antibacterial activity mediated by increased local pH and ion release, increasing osmotic pressure due to the exchange of alkaline ions with protons in solution in body fluid, making it a valuable adjunct in the chronic osteomyelitis treatment [14].

To address these gaps, the present study evaluated in vitro synergistic interactions between vancomycin, gentamicin, and ceftriaxone against two clinical *C. acnes* isolates representing infection-associated phylotypes, as well as a reference strain (ATCC 6919). Furthermore, we assessed the in vivo efficacy of these combinations, with or without a bioceramics (bioactive glass S53P4) supernatant, using the *Galleria mellonella* infection model, which has been proven to be a robust system for evaluating antimicrobial efficacy and host–pathogen dynamics [15].

## 2. Results

Antimicrobial susceptibility testing revealed uniformly low MICs across all *C. acnes* strains. For VA, MICs ranged from 0.25 to 0.5 μg/mL, with both clinical strains exhibiting MICs of 0.25 μg/mL, while the ATCC 6919 demonstrated a MIC of 0.5 μg/mL. For GEN, MICs were consistently higher in the clinical isolates (2 μg/mL) compared to ATCC 6919 (0.5 μg/mL). CTX showed the greatest variability, with MICs of 0.06 μg/mL for ATCC 6919, 0.125 μg/mL for strain 1, and 2 μg/mL for strain 2.

For clinical strain 1, partial synergistic effects were observed with the combination of CTX and GEN at two concentration pairs: 0.125 μg/mL CTX + 1 μg/mL GEN, and 0.06 μg/mL CTX + 1 μg/mL GEN (Table 1). For clinical strain 2, CTX and GEN resulted in multiple synergistic interactions, specifically at the following concentration pairs: 0.25 μg/mL CTX with 0.03, 0.06, or 0.125 μg/mL GEN; 0.125 μg/mL CTX with 0.25 or 0.5 μg/mL GEN; and 0.06 μg/mL CTX with 0.5 μg/mL GEN, as detailed in Table 2. The CKA demonstrated partial synergy between VA and CTX in *C. acnes* ATCC 6919, particularly at the combination of 0.25 μg/mL VA with 0.01 μg/mL CTX (Table 3). For all other isolates, as documented in Supplementary Materials, the combinations of VA with GEN, VA with CTX, and CTX with GEN demonstrated indifferent interactions, based on the calculated fractional inhibitory concentration (FIC) indices (Supplementary Materials, Tables S1–S4).

### 2.1. Optimal Concentration of C. acnes in the Galleria mellonella Infection Assay Using BAG Supernatant and Antimicrobials in a Synergistic Approach

Analysis of bacterial inoculation at different concentrations in the *G. mellonella* larval model revealed that a concentration corresponding to an optical density (OD_600_) of 3 resulted in a significant decrease in larval survival over the 5-day experimental period. The health and mortality index scoring system indicated high scores for the negative control group (saline), as larvae exhibited normal activity, cocoon formation, absence of melanization, and full survival throughout this study. Larvae inoculated with OD_600_ concentrations of 0.5, 1, 1.5, and 2 displayed high to moderate scores, with partial melanization observed; however, no significant differences were noted in other categories compared to the negative control, and larvae survived until pupation. In contrast, larvae exposed to an OD_600_ of 3 showed a progressive decline in health, culminating in complete mortality by day 3. Bacterial loads corresponding to an OD_600_ of 3 were estimated at 2.2 × 10^17^ CFU for ATCC strains, 5.5 × 10^14^ CFU for strain 1, and 1.3 × 10^15^ CFU for strain 2.

**Figure 1:**
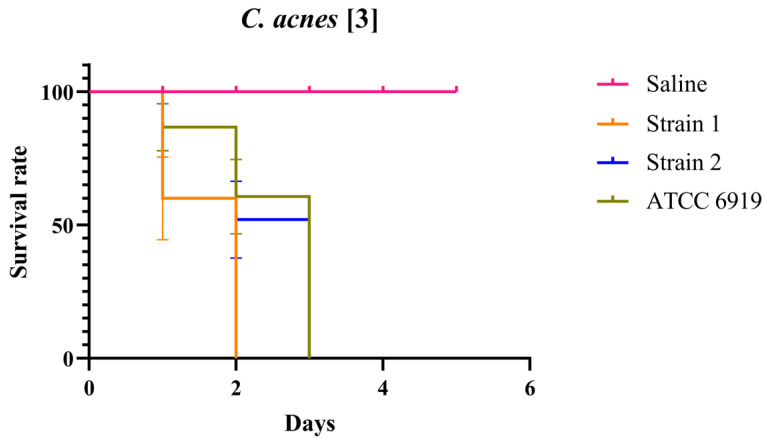
Survival graph of *G. mellonella* challenged with different strains of *C. acnes* at an OD_600_ of 3.

As demonstrated in Figure 1, larvae infected with clinical strain 1 exhibited mortality one day earlier than those infected with the other tested strains. Nevertheless, all strains induced mortality within the established experimental timeframe. Conversely, larvae in the negative control group remained viable throughout the 5-day observation period.

### 2.2. Efficacy and Synergism of Antibiotics and Interaction with BAG for C. acnes in the G. mellonella Infection Assay

**Figure 2:**
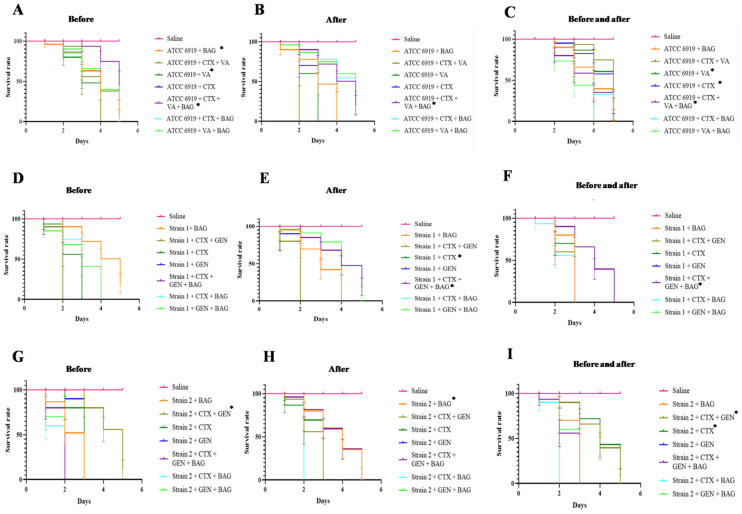
Survival percentage graph of *G. mellonella* challenged with different strains of *C. acnes* at an OD_600_ of 3. Treatments consisted of: (**A**) Larval group treated before ATCC 6919 strain challenge (**B**) Larval group treated after ATCC 6919 strain challenge; (**C**) Larval group treated before and after ATCC 6919 strain challenge; (**D**) Larval group treated before strain 1 challenge; (**E**) Larval group treated after strain 1 challenge; (**F**) Larval group treated before and after strain 1 challenge; (**G**) Larval group treated before strain 2 challenge; (**H**) Larval group treated after strain 2 challenge; (**I**) Larval group treated before and after strain 2 challenge. BAG = bioactive glass; CTX = ceftriaxone; VA = vancomycin; GEN = gentamicin; * refers to *p* < 0.001.

The *G. mellonella* infection assay, as depicted in Figure 2, demonstrated that antibiotic treatment regimens administered both pre- and post-infection resulted in enhanced larval survival for *C. acnes* ATCC 6919 and clinical strain 2 (Figure 2A,B,G,H). Although none of the therapeutic interventions completely prevented larval mortality, significant improvements in survival were observed compared to untreated controls. For strain ATCC 6919, treatments with VA, CTX, and the combination of CTX + VA + BAG markedly prolonged larval survival when administered both before and after infection (*p* < 0.001) (Figure 2C). For strain 2, the combinations of CTX + GEN and CTX alone yielded the most favorable outcomes within the same treatment group (*p* < 0.001) (Figure 2I). In contrast, for strain 1, prolonged larval survival was observed exclusively when CTX alone or in combination with GEN + BAG was administered post-infection (*p* < 0.001) (Figure 2E).

## 3. Discussion

To the best of our knowledge, this is among the few studies to systematically investigate the in vitro synergistic effects of vancomycin, ceftriaxone, and gentamicin against *C. acnes* using clinically relevant strains, complemented by in vivo validation through the *G. mellonella* infection model. Although all tested strains belonged to the same phylotype (IB), notable differences in antimicrobial susceptibility profiles were observed, emphasizing the considerable genetic and phenotypic variability within this clade. Notwithstanding, our findings revealed that either ceftriaxone with gentamicin or in combination of these antibiotics with BAG exhibited the most consistent synergism for strain 2 of *C. acnes*. In contrast, partial synergy was observed with the combination of ceftriaxone with gentamicin in strain 1, and vancomycin with ceftriaxone in ATCC 6919. Tiltnes et al. (2020), for instance, employed ceftriaxone at a dosage of 2 g/day to treat severe *C. acnes* infections, despite this approach remaining outside conventional treatment guidelines [16]. Other reports have suggested effective combination regimens, such as ceftriaxone associated with rifampicin or gentamicin [17,18,19], reinforcing the potential clinical applicability of the combinations tested in the present study.

Gentamicin, although not traditionally recommended for *C. acnes* monotherapy due to its variable efficacy and limited elution from antibiotic-loaded bone cement [20], demonstrated a potentiating effect when combined with ceftriaxone. These results are clinically relevant, especially in light of previous reports indicating minimal activity of gentamicin when used alone in this context [1]. Nevertheless, recent evidence has demonstrated that gentamicin, when used synergistically with other agents, can significantly impair *C. acnes* biofilm formation, achieving reductions of up to 93% in vitro [21]. This suggests that gentamicin’s role within combination therapies warrants reconsideration, particularly for prophylactic or adjunctive use in orthopedic infections.

The observed synergistic effects are likely attributable to the complementary mechanisms of action of ceftriaxone and gentamicin. While ceftriaxone inhibits bacterial cell wall synthesis, gentamicin binds to the 30S ribosome’s portion, inhibiting protein synthesis, resulting in a compounded antimicrobial effect [22]. Such dual-target strategies may be particularly advantageous during the initial phases of biofilm formation, when bacterial metabolic activity remains elevated and susceptibility to antimicrobials is enhanced [23]. Interestingly, a recently published meta-analysis evidenced the lack of documented resistance to vancomycin, gentamicin, and β-lactams in *C. acnes*, in contrast to higher rates of clindamycin, macrolides, and tetracyclines, reinforcing the importance of continuing assessment of resistance patterns of this pathogen in clinical practice [4]. Indeed, bone and joint infection caused by *C. acnes* usually shows a favorable susceptibility profile, especially for β-lactams, quinolones, and rifampicin [11]. Nevertheless, the variable responses observed in our study, even among strains of the same phylotype, suggest that phylotyping alone may be insufficient to guide targeted antimicrobial therapy. A more nuanced diagnostic approach integrating both genotypic and phenotypic data is, therefore, essential to optimize therapeutic outcomes in patients with orthopedic implant-associated infections.

The *G. mellonella* infection model proved to be a practical and informative platform for assessing the in vivo efficacy of antimicrobial combinations. In the present study, the steps to establish infection required a high bacterial inoculum, reflecting the inherently low virulence of *C. acnes* in systemic infection models [1]. Our results further revealed strain-dependent differences in pathogenicity and therapeutic response. Similar findings have been reported in other invertebrate models, such as the silkworm, where high inocula were likewise necessary to induce infection [24]. However, they primarily focused on assessing monotherapies at fixed high antimicrobial concentrations without exploring synergistic interactions. Thus, our study extends current knowledge by elucidating strain-specific responses to combinatorial antimicrobial strategies.

Interestingly, our study design was influenced by the results published by Mannala et al. [15] who successfully employed *G. mellonella* as an invertebrate model to assess *S. aureus* biofilm- and implant-associated infections. In their first approach, larval tolerance to implanted materials was primarily evaluated, followed by the development of biofilm and treatment responses. They reported biofilm formation on the implant surfaces, and while gentamicin had no observable effect on biofilm-associated bacteria, it significantly reduced the planktonic bacterial burden. In a subsequent study, the same group assessed the efficacy of antibiotic-loaded bone cement (ALBC) using the *G. mellonella* model. Among the tested formulations, only cement with vancomycin and gentamicin was able to significantly reduce the bacterial load up to the second day of infection [25]. Notably, this is the first study to investigate *C. acnes* in an antibiotic combination treatment with a biomaterial infection model using *G. mellonella*, highlighting a novel and promising approach for in vivo assessment of low-virulence pathogens. This in vivo approach provides a cost-effective and ethically acceptable alternative that enables the assessment of bacterial virulence and host-pathogen interactions in a dynamic infection setting. The absence of prior studies using *G. mellonella* to evaluate *C. acnes* in this context underscores the novelty and relevance of our findings.

Additionally, the inclusion of bioactive glass S53P4 in combination therapies offers a promising local adjuvant approach for managing implant-associated infections. This bioceramic has been extensively documented to possess both osteoconductive and antimicrobial properties, primarily mediated through the release of biologically active ions, such as calcium and silicon, which can inhibit bacterial growth while promoting tissue regeneration [25,26,27]. Despite this proven anti-infective mechanism of action, previous studies support the antimicrobial effect of this biomaterial without the need for additional antibiotic compounds. Moreover, its integration into local delivery systems may enhance the efficacy of antimicrobial combinations, especially in the context of implant-associated infections characterized by biofilm formation and limited vascularization. Future studies should aim to further elucidate the role of BAG in more complex in vivo models that more accurately replicate human bone and implant environments [28].

Several limitations of this study should be acknowledged. First, although the *G. mellonella* model offers advantages such as ethical acceptability, low cost, and high-throughput screening, its innate immune system—while analogous in some respects—lacks the complexity of mammalian adaptive immunity. This biological gap may limit the direct translatability of in vivo results to human infections. Second, only two *C. acnes* strains belonging to the same phylotype were evaluated. Given the known genetic and phenotypic heterogeneity among *C. acnes* phylotypes, the generalizability of the results remains limited. Third, the antibiotic release kinetics and diffusion dynamics observed in vitro may not fully replicate the local pharmacokinetics encountered in human bone or implant-associated infections. Moreover, while this study focused on antimicrobial efficacy, it did not directly quantify biofilm biomass or viability, which would provide complementary data on biofilm disruption.

Despite these limitations, the findings contribute to the growing body of evidence supporting precision-based antimicrobial strategies for the management of *C. acnes*-related orthopedic infections. Given the increasing reliance on prosthetic implants and the challenges posed by biofilm-associated pathogens, optimizing antimicrobial combinations and local delivery approaches is essential to improving clinical outcomes. Future translational research should focus on validating these synergistic effects in mammalian models, expanding strain diversity, and incorporating advanced techniques to better characterize biofilm behavior. Bridging the gap between experimental models and clinical practice is a critical step toward the effective implementation of novel therapeutic strategies in orthopedic infection management.

## 4. Materials and Methods

### 4.1. Bacterial Isolates

*C. acnes* clinical strains were previously sequenced by our group and analyzed in a previous study [11]. Therefore, for the present experiments, two representative phylotype IB clinical strains 1 and 2 were chosen, alongside the *C. acnes* reference strain ATCC 6919, which served as a control. All strains were preserved in triplicate cryotubes containing tryptic soy broth (TSB) (KASVI^®^, Sao Jose dos Pinhais, Brazil) supplemented with 20% glycerol (Synth^®^, Sao Paulo, Brazil) and one drop of defibrinated sheep blood, and stored at −20 °C. For experimental procedures, aliquots were subcultured onto sheep blood agar (Probac do Brasil^®^, Sao Paulo, Brazil) and Schaedler agar (Merck^®^, Darmstadt, Germany) plates, in triplicate. Cultures were incubated anaerobically at 37 °C for seven days using an Anaerobac^®^ anaerobiosis generator (Probac do Brasil^®^, São Paulo, Brazil).

### 4.2. Determination of Minimum Inhibitory Concentration (MIC) and Checkerboard Assay for Antimicrobial Treatment

The minimum inhibitory concentrations (MICs) of vancomycin (VA), gentamicin (GEN), and ceftriaxone (CTX) were determined for each strain using the broth microdilution method, following the guidelines established by the Brazilian Committee on Antimicrobial Susceptibility Testing [29]. The MIC was defined as the lowest concentration of the antimicrobial agent that completely inhibited visible bacterial growth. Interpretation of the results was performed in accordance with BrCAST recommendations [29].

Subsequently, a checkerboard assay was conducted to evaluate potential interactions between the selected antimicrobials. The concentration ranges for each agent were established based on their respective MIC values. Fractional inhibitory concentration (FIC) indices were calculated based on the absence of bacterial growth along the rows and columns corresponding to each antimicrobial combination, as previously described by Leber et al. (2016) [30].

Interpretation of the ΣFIC results was classified as follows:

- Synergy: ΣFIC ≤ 0.5, indicating a greater effect than that observed with the individual agents;
- Partial synergy: 0.5 < ΣFIC ≤ 1.0, indicating a weakly additive effect;
- Indifference: 1.0 < ΣFIC ≤ 4.0, indicating that the combination has a similar effect to that of the individual agents;
- Antagonism: ΣFIC > 4.0, indicating that the combination is less effective than the individual agents.

### 4.3. Bioactive Glass S53P4 (BAG) Preparation

The antimicrobial activity of bioactive bioglass S53P4 (<45 µm; BonAlive^®^ Biomaterials, Turku, Finland) was evaluated at a final concentration of 100 mg/mL. Granules were suspended in saline solution, vortexed for 60 s, and incubated at 37 °C for one day or until a pH 10 was reached. The supernatant was obtained by removing the liquid prior to use in the assays.

### 4.4. Galleria mellonella Infection Model

The selected *C. acnes* strains were standardized for inoculation based on optical density (OD_600_) to achieve final concentrations of 0.5, 1, 2, and 3, using an automated microplate reader (BioTek Epoch2^®^, Santa Clara, CA, USA). For each strain, five larvae were selected and distributed across three Petri dishes. All larvae weighed 250 ± 50 mg and were incubated at 37 °C for acclimatization prior to infection. An additional group of ten larvae served as the negative control and was maintained under the same conditions without bacterial challenge.

Experimental groups were inoculated with 10 μL of the respective bacterial suspension, injected into the last right posterior proleg. Following infection, larvae were incubated at 37 °C for five days. Larvae were considered dead upon exhibiting a change in coloration from pale yellow to dark brown or black, along with the absence of response to gentle tactile stimulation. At the end of the five-day observation period, survival data were compiled using Microsoft Excel and analyzed with GraphPad Prism, version 8.0.2 (GraphPad Software Inc., San Diego, CA, USA), for the generation of survival curves.

### 4.5. Infection Assay in Galleria mellonella Using BAG Supernatant and Antimicrobials in a Synergistic Approach

After establishing the optimal bacterial concentration for inducing larval mortality within five days, new larvae were employed to evaluate the in vivo effects of synergistic antimicrobial therapies and their combination with bioactive glass S53P4 (BAG). Bacterial suspensions of each *C. acnes* strain were adjusted to an OD_600_ of 3, and 10 μL of each suspension was injected into the last right proleg of each larva (*n* = 5 per group, in triplicate). Control groups included uninfected larvae injected with sterile saline. Three treatment protocols were tested, with 10 μL into the last left proleg of the larva at different time sets: Group 1 consisted of pre- and post-infection treatment analysis; Group 2 consisted of pre-infection treatment only; and Group 3 consisted of post-infection treatment only.

Due to MIC differences, treatment regimens for the ATCC 6919 strain consisted of VA (0.5 µg/mL), or CTX (0.06 µg/mL), a combination of VA and CTX (0.25 µg/mL + 0.01 µg/mL, respectively), BAG alone, and a triple combination (VA + CTX + BAG). For clinical strain 1, treatment regimens were CTX (0.125 µg/mL), GEN (2 µg/mL), a combination of CTX + GEN (0.125 µg/mL + 1 µg/mL, respectively), BAG alone, and a triple combination (CTX + GEN + BAG). For clinical strain 2, treatment regimens were CTX (2 µg/mL), GEN (0.25 µg/mL), a combination of CTX + GEN (0.06 µg/mL + 0.5 µg/mL, respectively), BAG alone, and a triple combination. Larvae were incubated at 37 °C and monitored daily for 5 days. Survival curves were analyzed using GraphPad Prism 8.0.2 (GraphPad Software Inc., San Diego, CA, USA). For survival analysis, a two-way ANOVA was performed.

## 5. Conclusions

This study provides novel insights into the synergistic potential of ceftriaxone and gentamicin—particularly when combined with bioactive glass S53P4—against *C. acnes*, using both clinical and reference strains and validated through an in vivo *G. mellonella* model. The results highlight that such combinations can significantly enhance antimicrobial activity, likely through complementary mechanisms of action and sustained local release, offering a promising strategy for the management of implant-associated infections caused by low-virulence pathogens. Furthermore, the strain-dependent variability in antimicrobial susceptibility, despite belonging to the same phylotype, underscores the importance of integrating genotypic and phenotypic analyses to guide targeted therapy. The use of *G. mellonella* as a model for *C. acnes* biomaterial-associated infection represents a cost-effective and ethically acceptable platform for preclinical assessment, bridging an important gap in experimental approaches for this pathogen. These findings support further exploration of antibiotic–biomaterial combinations in mammalian models and clinical settings, with the ultimate goal of optimizing prophylactic and therapeutic protocols for orthopedic implant-associated infections.

## Figures and Tables

**Table 1:** Checkerboard assay results for gentamicin and ceftriaxone against *C. acnes* clinical strain 1.

Interpretation	ΣFIC (FIC A + B)	Gentamicin		Ceftriaxone		Plate Well
		Gentamicin FIC	Concentration (μg/mL)	Ceftriaxone FIC	Concentration (μg/mL)	
Gentamicin MIC	Gentamicin MIC	Gentamicin MIC	0	Gentamicin MIC	0.125	A4
**Partial synergy**	0.625	0.5	1	1	0.125	B4
**Partial synergy**	0.625	0.5	1	1	0.125	C4
**Partial synergy**	0.625	0.5	1	1	0.125	D4
**Partial synergy**	0.625	0.5	1	1	0.125	E4
**Partial synergy**	0.625	0.5	1	1	0.125	F4
**Partial synergy**	0.56	0.5	1	0.48	0.06	F3
Indifferent	1.06	1	2	0.48	0.06	G3
Indifferent	1.03	1	2	0.24	0.03	G2
Ceftriaxone MIC	Ceftriaxone MIC	Ceftriaxone MIC	2	Ceftriaxone MIC	Ceftriaxone MIC	G1

MIC = minimum inhibitory concentration and FIC = fractional inhibitory concentration.

**Table 2:** Checkerboard assay results for ceftriaxone and gentamicin against *C. acnes* clinical strain 2.

Interpretation	ΣFIC (FIC A + B)	Gentamicin		Ceftriaxone		Plate Well
		Gentamicin FIC	Concentration (μg/mL)	Ceftriaxone FIC	Concentration (μg/mL)	
Gentamicin MIC	Gentamicin MIC	Gentamicin MIC	0	Gentamicin MIC	0.25	A5
**Synergy**	0.265	0.015	0.03	1	0.25	B5
**Synergy**	0.28	0.03	0.06	1	0.25	C5
**Synergy**	0.31	0.06	0.125	1	0.25	D5
**Synergy**	0.25	0.125	0.25	0.5	0.125	D4
**Synergy**	0.375	0.25	0.5	0.5	0.125	E4
**Synergy**	0.31	0.25	0.5	0.24	0.06	E3
Indifferent	1.06	1	2	0.24	0.06	F3
Indifferent	1.06	1	2	0.24	0.06	G3
Indifferent	1.03	1	2	0.12	0.03	G2
Ceftriaxone MIC	Ceftriaxone MIC	Ceftriaxone MIC	2	Ceftriaxone MIC	Ceftriaxone MIC	G1

MIC = minimum inhibitory concentration and FIC = fractional inhibitory concentration.

**Table 3:** Checkerboard assay results for vancomycin and ceftriaxone against *C. acnes* ATCC 6919.

Interpretation	ΣFIC (FICA + B)	Ceftriaxone		Vancomycin		Plate Well
		Ceftriaxone FIC	Concentration (μg/mL)	Vancomycin FIC	Concentration (μg/mL)	
Vancomycin MIC	Vancomycin MIC	Vancomycin MIC	0	Vancomycin MIC	0.5	A11
Indifferent	1.1	0.1	0.006	1	0.5	B11
Indifferent	1.01	0.01	0.001	1	0.5	C11
Indifferent	1.04	0.04	0.0025	1	0.5	D11
Indifferent	1.08	0.08	0.005	1	0.5	E11
Indifferent	1.16	0.16	0.01	1	0.5	F11
**Partial synergy**	0.66	0.16	0.01	0.5	0.25	F10
Indifferent	1.5	1	0.06	0.5	0.25	G10
Indifferent	1.25	1	0.06	0.25	0.125	G9
Indifferent	1.12	1	0.06	0.12	0.06	G8
Indifferent	1.06	1	0.06	0.06	0.03	G7
Indifferent	1.03	1	0.06	0.03	0.01	G6
Indifferent	1.01	1	0.06	0.01	0.005	G5
Indifferent	1.005	1	0.06	0.005	0.0025	G4
Indifferent	1.002	1	0.06	0.002	0.001	G3
Indifferent	1.012	1	0.06	0.012	0.006	G2
Ceftriaxone MIC	Ceftriaxone MIC	Ceftriaxone MIC	0.06	Ceftriaxone MIC	Ceftriaxone MIC	G1

MIC = minimum inhibitory concentration and FIC = fractional inhibitory concentration.

## Data Availability

The data are available from the corresponding author upon reasonable request.

## Supplementary Materials

The following supporting information can be downloaded at https://www.mdpi.com/article/10.3390/antibiotics14090923/s1, Table S1. Checkerboard assay results for vancomycin and gentamicin against *C. acnes* ATCC 6919; Table S2. Checkerboard assay results for vancomycin and ceftriaxone against *C. acnes* clinical strains 1 and 2; Table S3. Checkerboard assay results for vancomycin and gentamicin against *C. acnes* clinical strain 1; Table S4. Checkerboard assay results for vancomycin and gentamicin against *C. acnes* clinical strain 2, and ceftriaxone and gentamycin against *C. acnes* ATCC 6919.

## Abbreviations

The following abbreviations are used in this manuscript:

VA	Vancomycin
GEN	Gentamicin
CTX	Ceftriaxone
BAG	Bioactive glass S53P4
MICs	Minimum inhibitory concentrations
FICIs	Fractional inhibitory concentration indices
TSB	Tryptic soy broth
ALBC	Antibiotic-loaded bone cement

## References

[1] Thoraval L., Varin-Simon J., Ohl X., Velard F., Reffuveille F., Tang-Fichaux M. (2025). *Cutibacterium acnes* and its complex host interaction in prosthetic joint infection: Current insights and future directions. Res. Microbiol..

[2] Tafin U.F., Trampuz A., Corvec S. (2013). In vitro emergence of rifampicin resistance in *Propionibacterium acnes* and molecular characterization of mutations in the *rpoB* gene. J. Antimicrob. Chemother..

[3] Aoki S., Nakase K., Hayashi N., Noguchi N. (2019). Transconjugation of *erm(X)* conferring high-level resistance of clindamycin for *Cutibacterium acnes*. J. Med. Microbiol..

[4] Beig M., Shirazi O., Ebrahimi E., Banadkouki A.Z., Golab N., Sholeh M. (2024). Prevalence of antibiotic-resistant *Cutibacterium acnes* (formerly *Propionibacterium acnes*) isolates, a systematic review and meta-analysis. J. Glob. Antimicrob. Resist..

[5] McDowell A., Nagy I., Magyari M., Barnard E., Patrick S., Zhou D. (2013). The opportunistic pathogen *Propionibacterium acnes*: Insights into typing, human disease, clonal diversification and CAMP factor evolution. PLoS ONE.

[6] Spittaels K.-J., Ongena R., Zouboulis C.C., Crabbé A., Coenye T. (2020). *Cutibacterium acnes* Phylotype I and II Strains Interact Differently with Human Skin Cells. Front. Cell. Infect. Microbiol..

[7] Salar-Vidal L., Achermann Y., Aguilera-Correa J.-J., Poehlein A., Esteban J., Brüggemann H., on behalf of the ESCMID Study Group for Implant-Associated Infections (ESGIAI) (2021). Genomic Analysis of *Cutibacterium acnes* Strains Isolated from Prosthetic Joint Infections. Microorganisms.

[8] Cavallo I., Sivori F., Truglio M., De Maio F., Lucantoni F., Cardinali G., Pontone M., Bernardi T., Sanguinetti M., Capitanio B. (2022). Skin dysbiosis and *Cutibacterium acnes* biofilm in inflammatory acne lesions of adolescents. Sci Rep..

[9] van Vugt T.A.G., Arts J.J., Geurts J.A.P. (2019). Antibiotic-Loaded Polymethylmethacrylate Beads and Spacers in Treatment of Orthopedic Infections and the Role of Biofilm Formation. Front. Microbiol..

[10] Hoch A., Fritz Y., Dimitriou D., Bossard D.A., Fucentese S.F., Wieser K., Achermann Y., Zingg P.O. (2023). Treatment outcomes of patients with *Cutibacterium acnes*-positive cultures during total joint replacement revision surgery: A minimum 2-year follow-up. Arch. Orthop. Trauma Surg..

[11] Kurihara M.N.L., Santos I.N.M., Eisen A.K.A., Caleiro G.S., de Araújo J., de Sales R.O., Pignatari A.C., Salles M.J. (2023). Phenotypic and Genotypic Characterization of *Cutibacterium acnes* Isolated from Shoulder Surgery Reveals Insights into Genetic Diversity. Microorganisms.

[12] Kusejko K., Auñón Á., Jost B., Natividad B., Strahm C., Thurnheer C., Pablo-Marcos D., Slama D., Scanferla G., Uckay I. (2021). The Impact of Surgical Strategy and Rifampin on Treatment Outcome in *Cutibacterium* Periprosthetic Joint Infections. Clin. Infect. Dis..

[13] Saltiel G., Meyssonnier V., Kerroumi Y., Heym B., Lidove O., Marmor S., Zeller V. (2022). *Cutibacterium acnes* Prosthetic Joint Infections: Is Rifampicin-Combination Therapy Beneficial?. Antibiotics.

[14] Tanwar Y.S., Ferreira N. (2020). The role of bioactive glass in the management of chronic osteomyelitis: A systematic review of literature and current evidence. Infect. Dis..

[15] Mannala G.K., Rupp M., Alagboso F., Kerschbaum M., Pfeifer C., Sommer U., Kampschulte M., Domann E., Alt V. (2021). *Galleria mellonella* as an alternative in vivo model to study bacterial biofilms on stainless steel and titanium implants. ALTEX.

[16] Tiltnes T.S., Kehrer M., Hughes H., Morris T.E., Justesen U.S. (2020). Ceftriaxone treatment of spondylodiscitis and other serious infections with *Cutibacterium acnes*. J. Antimicrob. Chemother..

[17] Charles P., Hot A., Ou P., Carbonnelle E., Sidi D., Nassif X., Lortholary O. (2007). *Propionibacterium acnes* endocarditis in an adolescent boy suffering from a congenital cardiopathy. Pediatr. Infect. Dis. J..

[18] Kurz M., Kaufmann B.A., Baddour L.M., Widmer A.F. (2014). *Propionibacterium acnes* prosthetic valve endocarditis with abscess formation: A case report. BMC Infect. Dis..

[19] Vinod A., Listopadzki T., Kohut K., Pavlesen S., Crane J., Feng L., Duquin T., DiPaola M. (2023). An in vitro analysis of various antibiotic cement combinations against *Cutibacterium acnes*. Semin. Arthroplast. JSES.

[20] Kim Y.-G., Lee J.-H., Kim S., Park S., Kim Y.-J., Ryu C.-M., Seo H.W., Lee J. (2024). Inhibition of Biofilm Formation in *Cutibacterium acnes*, *Staphylococcus aureus*, and *Candida albicans* by the Phytopigment Shikonin. Int. J. Mol. Sci..

[21] Usman M., Markus A., Fatima A., Aslam B., Zaid M., Khattak M., Bashir S., Masood S., Rafaque Z., Dasti J.I. (2023). Synergistic Effects of Gentamicin, Cefepime, and Ciprofloxacin on Biofilm of *Pseudomonas aeruginosa*. Infect. Drug Resist..

[22] Hawas S., Verderosa A.D., Totsika M. (2022). Combination Therapies for Biofilm Inhibition and Eradication: A Comparative Review of Laboratory and Preclinical Studies. Front. Cell. Infect. Microbiol..

[23] Matsumoto Y., Tateyama Y., Sugita T. (2021). Evaluation of Antibacterial Drugs Using Silkworms Infected by *Cutibacterium acnes*. Insects.

[24] Zhao Y., Mannala G.K., Youf R., Rupp M., Alt V., Riool M. (2024). Development of a *Galleria mellonella* Infection Model to Evaluate the Efficacy of Antibiotic-Loaded Polymethyl Methacrylate (PMMA) Bone Cement. Antibiotics.

[25] Hoppe A., Güldal N.S., Boccaccini A.R. (2011). A review of the biological response to ionic dissolution products from bioactive glasses and glass-ceramics. Biomaterials.

[26] Zhang D., Leppäranta O., Munukka E., Ylänen H., Viljanen M.K., Eerola E., Hupa M., Hupa L. (2009). Antibacterial effects and dissolution behavior of six bioactive glasses. J. Biomed. Mater. Res. Part A.

[27] Gerhardt L.C., Boccaccini A.R. (2010). Bioactive Glass and Glass-Ceramic Scaffolds for Bone Tissue Engineering. Materials.

[28] Baino F., Hamzehlou S., Kargozar S. (2018). Bioactive Glasses: Where Are We and Where Are We Going?. J. Funct. Biomater..

[29] Brazilian Committee on Antimicrobial Susceptibility Testing—BrCAST (2025). Tabelas de Pontos de Corte Para Interpretação de CIMs e Diâmetros de Halos.

[30] Leber A.L. (2016). Clinical Microbiology Procedures Handbook.

