# Supplementary material for: Synergistic Antimicrobial Activity of Vancomycin, Ceftriaxone, and Gentamicin Against Cutibacterium acnes Strains: An In Vitro Checkerboard Analysis and In Vivo Interaction with Bioactive Glass Using Galleria mellonella
Source: Antibiotics (Basel). 2025 Sep 12;14(9):923. doi: 10.3390/antibiotics14090923 (PMC12466660; doi:10.3390/antibiotics14090923)
Supplement: Supplementary file 1 [file antibiotics-14-00923-s001.zip › antibiotics-3850116-supplementary.pdf]

antibiotics

MDPI

Article

Synergistic Antimicrobial Activity of Vancomycin, Ceftriaxone, and Gentamicin Against *Cutibacterium acnes* Strains: An In Vitro Checkerboard Analysis and In Vivo Interaction with Bioactive Glass Using *Galleria mellonella*

Mariana Neri Lucas Kurihara [1], Isabelle Frois Brasil [1], Mayara Muniz de Andrade Silva [1] and Mauro Jose Salles [1,2,3,*]

[1] Special Laboratory of Clinical Microbiology (LEMC), Department of Medicine, Division of Infectious Diseases, Escola Paulista de Medicina (EPM), Universidade Federal de São Paulo (UNIFESP), São Paulo 04024-002, Brazil; mariana.kurihara@unifesp.br (M.N.L.K.); isabelle.brasil@unifesp.br (I.F.B.); mmasilva@unifesp.br (M.M.d.A.S.)
[2] Musculoskeletal Infection Group, Department of Orthopedics and Traumatology, Escola Paulista de Medicina (EPM), Universidade Federal de São Paulo (UNIFESP), São Paulo 04024-002, Brazil
[3] Infectious Disease Discipline, Faculdade de Ciências Médicas da Santa Casa de São Paulo, São Paulo 01224-001, Brazil
* Correspondence: salles.infecto@gmail.com

Supplementary tables

Supplementary Table S1. Checkerboard assay results for vancomycin and gentamicin against *C. acnes* ATCC 6919.

Plate well	Vancomycin		Gentamicin		ΣFIC (FIC A+B)	Interpretation
	Concentration (µg/ml)	Vancomycin FIC	Concentration (µg/ml)	Gentamicin FIC		
A4	0.5	Vancomycin MIC	0	Vancomycin MIC	Vancomycin MIC	Vancomycin MIC
B4	0.5	1	2	1	2	Indifferent
C4	0.5	1	2	1	2	Indifferent
D4	0.5	1	2	1	2	Indifferent
E4	0.5	1	2	1	2	Indifferent
F4	0.5	1	2	1	2	Indifferent
G4	0.5	1	2	1	2	Indifferent
G3	0.25	0.5	2	1	1.5	Indifferent
G2	0.125	0.25	2	1	1.25	Indifferent
G1	Gentamicin MIC	Gentamicin MIC	2	Gentamicin MIC	Gentamicin MIC	Gentamicin MIC

MIC= minimum inhibitory concentration, FIC= fractional inhibitory concentration.

Supplementary Table S2. Checkerboard assay results for vancomycin and ceftriaxone against *C. acnes* clinical strains 1 and 2.

Plate well	Vancomycin		Ceftriaxone		ΣFIC (FIC A+B)	Interpretation
	Concentration (µg/ml)	Vancomycin FIC	Concentration (µg/ml)	Ceftriaxone FIC		
A11	0.5	Vancomycin MIC	0	Vancomycin MIC	Vancomycin MIC	Vancomycin MIC

 https://doi.org/10.3390/ antibiotics14090923

B11	0.5	1	0.006	0.1	1.1	Indifferent
C11	0.5	1	0.001	0.016	1.016	Indifferent
D11	0.5	1	0.0025	0.04	1.04	Indifferent
E11	0.5	1	0.005	0.08	1.08	Indifferent
F11	0.5	1	0.01	0.16	1.16	Indifferent
G11	0.5	1	0.03	0.5	1.5	Indifferent
H11	0.5	1	0.06	1	2	Indifferent
H10	0.25	0.5	0.06	1	1.5	Indifferent
H9	0.125	0.25	0.06	1	1.25	Indifferent
H8	0.06	0.125	0.06	1	1.125	Indifferent
H7	0.03	0.06	0.06	1	1.06	Indifferent
H6	0.01	0.03	0.06	1	1.03	Indifferent
H5	0.005	0.01	0.06	1	1.01	Indifferent
H4	0.0025	0.005	0.06	1	1.005	Indifferent
H3	0.001	0.0025	0.06	1	1.0025	Indifferent
H2	0.0006	0.001	0.06	1	1.001	Indifferent
H1	Ceftriaxone MIC	Ceftriaxone MIC	0.06	Ceftriaxone MIC	Ceftriaxone MIC	Ceftriaxone MIC

MIC= minimum inhibitory concentration, FIC= fractional inhibitory concentration.

Supplementary Table S3. Checkerboard assay results for vancomycin and gentamicin against *C. acnes* clinical strain 1.

Plate well	Vancomycin		Gentamicin		ΣFIC (FIC A+B)	Interpretation
	Concentration (µg/ml)	Vancomycin FIC	Concentration (µg/ml)	Gentamicin FIC		
A4	0.5	Vancomycin MIC	0	Vancomycin MIC	Vancomycin MIC	Vancomycin MIC
B4	0.5	1	2	1	2	Indifferent
C4	0.5	1	2	1	2	Indifferent
D4	0.5	1	2	1	2	Indifferent
E4	0.5	1	2	1	2	Indifferent
F4	0.5	1	2	1	2	Indifferent
G4	0.5	1	2	1	2	Indifferent
G3	0.25	0.5	2	1	1.5	Indifferent
G2	0.125	0.25	2	1	1.25	Indifferent
G1	Gentamicin MIC	Gentamicin MIC	2	Gentamicin MIC	Gentamicin MIC	Gentamicin MIC

MIC= minimum inhibitory concentration, FIC= fractional inhibitory concentration.

Supplementary Table S4. Checkerboard assay results for vancomycin and gentamicin against *C. acnes* clinical strain 2 and ceftriaxone and gentamycin against *C. acnes ATCC 6919.*

Plate well	Vancomycin		Gentamicina		ΣFIC (FIC A + B)	Interpretation
	Concentration (µg/ml)	Vancomycin FIC	Concentration (µg/ml)	Gentamicin FIC		
A4	0.5	Vancomycin MIC	0	Vancomycin MIC	Vancomycin MIC	Vancomycin MIC
B4	0.5	1	2	1	2	Indifferent
C4	0.5	1	2	1	2	Indifferent
D4	0.5	1	2	1	2	Indifferent
E4	0.5	1	2	1	2	Indifferent
F4	0.5	1	2	1	2	Indifferent
G4	0.5	1	2	1	2	Indifferent

H4	0.5	0.5	2	1	1.5	Indifferent
H3	0.25	0.5	2	1	1.5	Indifferent
H2	0.125	0.25	2	1	1.25	Indifferent
H1	Gentamicin MIC	Gentamicin MIC	2	Gentamicin MIC	Gentamicin MIC	Gentamicin MIC

MIC= minimum inhibitory concentration, FIC= fractional inhibitory concentration.
